# Trends in the Proportion of Female Speakers at Medical Conferences in the United States and in Canada, 2007 to 2017

**DOI:** 10.1001/jamanetworkopen.2019.2103

**Published:** 2019-04-12

**Authors:** Shannon M. Ruzycki, Sarah Fletcher, Madalene Earp, Aleem Bharwani, Kirstie C. Lithgow

**Affiliations:** 1 Division of General Internal Medicine, Department of Medicine, University of Calgary, Calgary, Alberta, Canada; 2 Faculty of Medicine, University of British Columbia, Vancouver, British Columbia, Canada; 3 W21C Research and Innovation Centre, Cumming School of Medicine, University of Calgary, Calgary, Alberta, Canada; 4 Division of Endocrinology and Metabolism, Department of Medicine, University of Calgary, Calgary, Alberta, Canada

## Abstract

**Importance:**

Gender equity is a prominent issue in the medical profession. Representation of female physicians at academic meetings has been identified as an important component of gender equity; however, this topic has not been systematically assessed.

**Objective:**

To determine the trend during the last decade in the proportion of speakers who were women at major academic medical conferences held in Canada and in the United States.

**Design, Setting, and Participants:**

A cross-sectional analysis was conducted examining the gender of speakers listed in meeting programs of medical conferences held in Canada and in the United States in 2007 and from 2013 through 2017. Eligible conferences were identified using a sensitive search strategy, and a previously validated tool was used to analyze each meeting speaker list and to assign a proportion of female speakers. Conferences held in English language, hosted in Canada or the United States, and targeted to a physician audience with 100 or more attendees were included. The comparison group was active physicians in Canada and in the United States.

**Main Outcomes and Measures:**

The mean of the proportion of female speakers at each conference per year.

**Results:**

In total, 181 conferences with 701 individual meetings were analyzed, including 100 medical and 81 surgical specialty conferences. The proportion of women ranged from 0% to 82.6% of all speakers. The mean (SD) proportion of female conference speakers for all meetings analyzed significantly increased from 24.6% (14.6%) for 40 meetings in 2007 to 34.1% (15.1%) for 181 meetings in 2017 (*P* < .001). The mean proportion of female speakers at medical specialty conferences was 9.8% higher (SE, 1.9%; *P* < .001) than the mean proportion of female speakers at surgical specialty conferences for all years analyzed. The mean proportion of female speakers at conferences was similar to the mean proportion of active female physicians across all specialties in the United States and in Canada for all years analyzed.

**Conclusions and Relevance:**

Although our findings indicate that the proportion of female speakers at medical conferences increased during the last decade, women continue to be underrepresented. Speaker invitation and selection at conferences represent important opportunities to influence gender equity within medicine.

## Key Points

**Question:**

What were the proportions of female speakers at academic medical conferences held in the United States and in Canada during the last decade?

**Findings:**

In this cross-sectional analysis of 181 medical conferences in 2007 and from 2013 through 2017, the proportions of female speakers significantly increased from 24.6% to 34.1%. These proportions were similar to the percentages of practicing physicians who were women during the same time frame.

**Meaning:**

Although the proportion of female speakers has increased during the last decade, women are underrepresented at medical conferences.

## Teaser

This cross-sectional study examines the proportion of female speakers at major academic medical conferences held in Canada and in the United States during the last decade.

## Introduction

Gender equity in medicine is a prominent topic of medical editorials, scientific literature, and mass media. Gender disparity in the medical profession has been well documented,^1,2,3^ including evidence for inequities in evaluation, hiring, compensation, harassment, promotion, and advancement.^1,3,4,5,6,7,8,9,10,11^ Contributors to gender inequity in medicine include implicit and explicit bias, cultural factors, unsupportive work environments, and representation of women in medicine.^1,2,4,6,11,12,13,14^

Visibility or representation of female physicians is an important facet of gender equity.^15,16^ Evidence from social science literature suggests that higher female representation leads to lower gender bias in the community.^17^ In academic settings, higher numerical representation of women correlates with improved outcomes, including improved career satisfaction and retention, for women and other minority groups.^15^ In medicine, a lack of role models and mentors has been identified as a barrier to advancement in surgical specialties and academic internal medicine that disproportionately affects female physicians.^1,6^ Underrepresentation occurs when the number of female physicians in visible positions is less than the proportion expected based on the number of female physicians overall.

Although women make up approximately 33% of the physician workforce in the United States and 42% in Canada, they are variably underrepresented in academia.^18,19,20^ Approximately one-third of academic physicians in Canada and the United States are women, and the proportion varies greatly by specialty^1,5^; only 8% of academic surgeons and 16.5% of academic cardiologists are women.^21^ Female scientists have fewer total publications and are less likely than their male colleagues to be listed as first author, even when first authorship is shared by coauthors of different sexes.^21,22,23^ Women are underrepresented as speakers at medical grand rounds and, when they do present, are less often addressed by their professional title than male presenters are.^12,24,25^

Although female representation at academic meetings has been identified as an important gender equity issue, the proportion of conference speakers who are women has not yet been systematically measured across different medical subspecialties, to our knowledge. Presenting at a medical conference is an opportunity for career advancement for female physicians and also represents an opportunity for trainees and colleagues to see female leaders in academia as role models.^15,16^

Underrepresentation of female physicians has been described at critical care conferences but has not been evaluated in other medical specialties.^20,26^ The phenomenon of all-male panels at scientific meetings recently gained attention on social media via the hashtag “NoMoreManels” and has prompted appeals to improve the gender balance in speaker programs.^16,27,28,29^ In this study, we aimed to measure the proportion of female speakers at medical conferences and to characterize trends in female representation between 2007 and 2017.

## Methods

### Data Sources and Search Strategy

We consulted a librarian to develop a systematic search strategy to identify relevant medical conferences. Conferences were identified using Web of Science Conference abstract databases by searching with key words developed using a list of medical specialties (searched February 18, 2018; search terms given in the eAppendix in the Supplement). The search was filtered for English-language conferences held in Canada or the United States. Additional conferences were solicited by email from program directors and chief residents in all accredited medical specialties.^30^ *Conference* refers to the host society or association, and *meeting* refers to the event held by the conference in a specific year. This report follows the Strengthening the Reporting of Observational Studies in Epidemiology (STROBE) reporting guidelines for reporting the results and the Preferred Reporting Items for Systematic Reviews and Meta-analyses (PRISMA) reporting guidelines when describing the search methods and results. The data analyzed were publicly available on conference websites, and therefore our institutional ethics review board waived the need for a review of this project and for obtaining participant informed consent.

### Inclusion and Exclusion Criteria

Conferences held outside Canada and the United States were excluded owing to potential cultural differences that may influence the gender of selected conference speakers. Non–English-language conferences were excluded. Conferences that did not have a 2017 meeting, were a chapter event of a larger society, were not held annually, were not intended for a primarily physician audience, or had fewer than 100 attendees were excluded. These criteria were intended to limit our analysis to major meetings without overrepresenting conferences with multiple events per year. To assess trends in the proportion of female speakers over time, we included 2007 data and 2013 through 2017 data when the conference program was available.

### Conference Selection and Data Extraction

Each identified conference was screened for eligibility by 2 independent reviewers (S.M.R. and K.C.L.), who applied the inclusion and exclusion criteria to the conference title. The conference website was then assessed in detail for inclusion by both reviewers. Disagreements were adjudicated by a third reviewer (A.B.).

The meeting program or faculty lists from eligible conferences were downloaded from the conference website or requested from the host association via email. Names were extracted from the meeting programs for 2007 and for 2013 through 2017, if available. When known, speakers who presented more than once at each conference were listed for each credit in the program to fully represent the gender balance at each conference. When specified, speakers who only presented posters were excluded.

### Data Analysis

Each list of names was analyzed using the Gender Balance Assessment Tool (GBAT)^31,32^ to assign a proportion of female speakers at that meeting during that year. The GBAT is a validated web-based tool that can identify names in a text-based document and assign a probabilistic prediction that the first name belongs to a female.^31^ The GBAT uses the genderize.io algorithm to assign each name a probability of belonging to a gender on the basis of social media data.^31,33^ The GBAT then aggregates the probabilities to return a likely proportion of female names in the document.^31^ The GBAT returns a single value: the proportion of names that are likely to be female, not the absolute number of female speakers at each conference. All conferences were weighted equally regardless of the number of attendees or presenters. All analyses were performed using the proportion of female speakers from each included meeting. The GBAT does not provide the number of names analyzed or a confidence interval.

Compared with hand-coding of names, the GBAT is more cost-effective, faster, incrementally adaptable to trends in names, and multinational and assigns a probability of gender rather than make a dichotomous assignment.^31,33^ Validation of the GBAT has demonstrated an area under the curve of 0.93 and a net error rate of 1.42% that usually overestimates the probability of a name being female.^31^ The GBAT is unable to assign gender to names that begin with initials.^31^

### Comparison Group

The comparison group was the mean proportion of practicing physicians in the United States and in Canada who were women in each specialty in 2007, 2010, 2013, and 2015 (the last year for which such data were available in the United States). This proportion was calculated by determining the proportion of practicing female physicians in each specialty and calculating the mean of those proportions for each year. This calculation was performed so that female representation in each specialty contributed equally to the comparison group regardless of specialty size, which aligned with our methods for determining the mean proportion of female speakers for each specialty. The mean proportions of women for all specialties and for medical specialties were similar to the actual percentage of women for these groups. The mean proportion of female surgeons differed from the absolute percentage of women in surgical specialties by approximately 7% per year; this difference may be attributable to the low proportion of women in numerous, smaller surgical specialties (Table 1).

**Table 1.** Female Physicians in All Specialties, Medical Specialties, and Surgical Specialties per Year in Canada and in the United States

Specialty	Year			
	2007	2010	2013	2015
All				
Total No. of physicians	828 602	867 665	903 395	938 505
No. of female physicians	236 852	266 730	297 577	322 817
Mean proportion female, %	26.1	28.5	30.6	32.4
Absolute proportion female, %	28.6	30.7	32.9	34.4
Medical				
Total No. of physicians	669 614	707 326	742 064	775 171
No. of female physicians	206 087	232 159	258 719	280 520
Mean proportion female, %	30.4	32.8	34.8	37.6
Absolute proportion female, %	30.8	32.8	34.9	36.2
Surgical				
Total No. of physicians	150 695	151 578	151 890	153 568
No. of female physicians	29 217	32 760	36 681	39 744
Mean proportion female, %	14.8	16.8	18.4	18.3
Absolute proportion female, %	19.4	21.6	24.2	25.9

### Statistical Analysis

The Cohen κ coefficient was used to determine agreement of conference inclusion between the 2 reviewers (S.M.R. and K.C.L.). The mean and SD were calculated using the proportion of speakers who were women at each conference for all conferences, medical conferences, and surgical conferences. The change in the proportion of female conference speakers over time (per year) was analyzed using a linear mixed-effects model (LMM), with individual conferences (random effects, assuming random intercepts with fixed mean) and clinical area (surgical or medical; fixed effect) specified in the model as covariates. The year was considered a fixed effect. The LMM analysis was used because it accounts for multiple responses from the same conference being expected to be more similar than responses from different conferences. Furthermore, LMM analysis permits missing data. The LMM analyses were performed with R (R Foundation for Statistical Computing, version 3.3.4) using the lme4 package.^34^ The *P* values were obtained with the lmerTest package.^35^ All tests were 2-sided, and *P* < .05 defined statistical significance.

## Results

### Search Outcome

**Figure 1.**
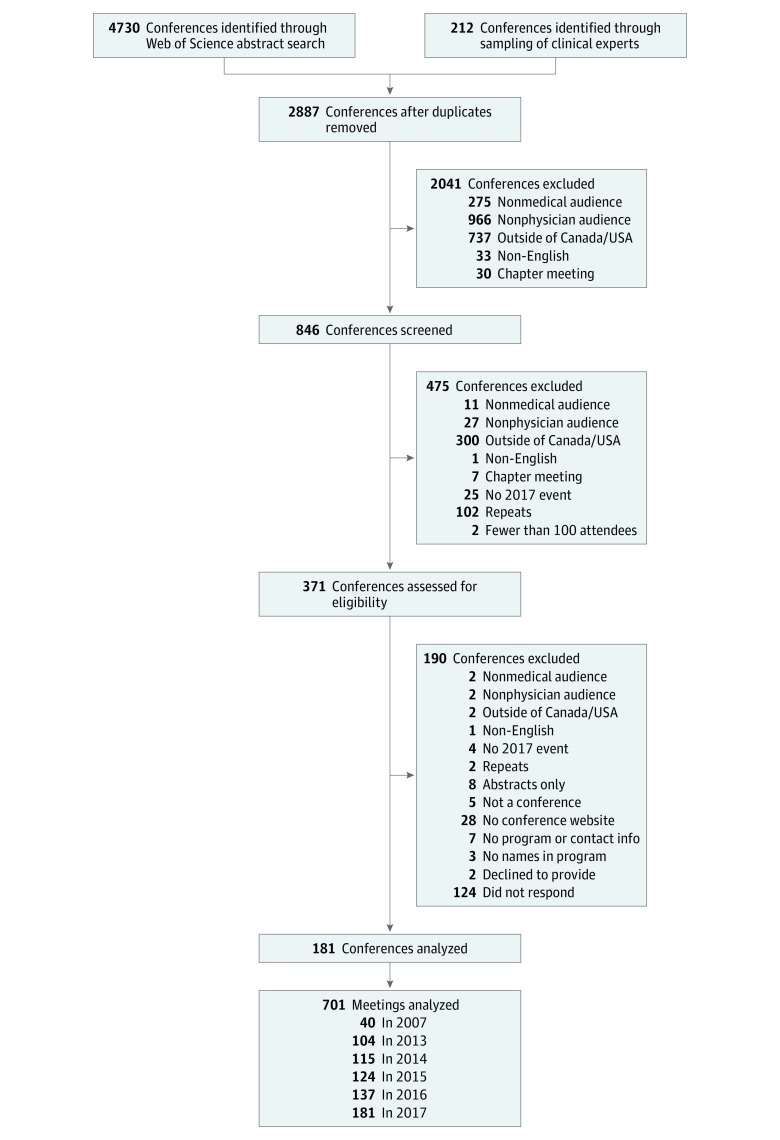
PRISMA Diagram of the Conference Search Strategy

The search strategy identified 4942 conferences. After removing duplicates, 2887 conferences were screened, of which 2041 did not meet inclusion criteria. The conference website was reviewed for 846 conferences, and 371 met full inclusion criteria (κ for agreement between the 2 reviewers, 0.805). Of those, 701 meeting programs were available from 181 conferences, which included 100 medical and 81 surgical specialty conferences. (Figure 1; eTable 1 in the Supplement). Meeting data from the 181 conferences were incomplete for 141 conferences (78.0%) in 2007, 77 (42.6%) in 2013, 66 (36.5%) in 2014, 57 (31.5%) in 2015, and 45 (24.9%) in 2016. Identification of 2017 data was an inclusion requirement and therefore available for 100% of conferences (Table 2; eTable 2 in the Supplement). The LMM statistical analysis accounts for missing data in effect estimates.

**Table 2.** Characteristics of Conferences Included in the Final Analysis

Characteristic	No. of Meetings	Female Speakers, Mean (SD), %
All conferences	181	
Year		
2007	40	24.6 (14.6)
2013	104	28.5 (14.1)
2014	115	30.6 (14.2)
2015	124	32.9 (15.7)
2016	137	33.5 (15.0)
2017	181	34.1 (15.0)
Total meetings	701	31.8 (15.0)
Host country		
United States	155	31.1 (15.3)
Canada	26	35.5 (12.8)

### Female Conference Speakers

**Figure 2.**
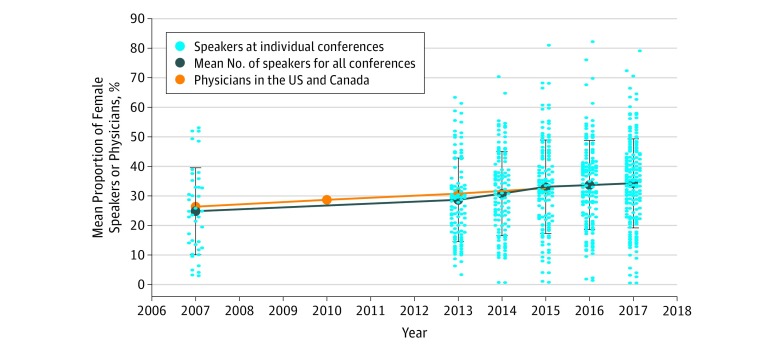
Mean Proportion of Female Speakers at Conference Meetings per Year and Proportion of Female Physicians, in the United States and Canada.

Between 2007 and 2017, the mean (SD) proportion of conference speakers who were female significantly increased from 24.6% (14.6%) for 40 meetings in 2007 to 34.1% (15.1%) for 181 meetings in 2017 (*P* < .001) (Figure 2). There was a wide range (0%-82.6%) in the proportion of female speakers for all meetings included. There was a 0.97% increase per year in the mean proportion of female speakers in this time frame (SE, 0.13%, *P* < .001). This rate of increase in the proportion of female speakers was not significantly different between medical specialty and surgical specialty conferences. In addition, 82 meetings (12%) had more than 50% female speakers. Between 2007 and 2015, the mean (SD) proportion of female physicians practicing in the United States and in Canada also increased, from 26.1% (13.1%) to 32.4% (15.2%) (data not available for 2016 and 2017).^18,19,36,37,38^ The proportions of practicing female physicians were similar to the proportions of female conference speakers for all years in which data were available (Figure 2).

**Figure 3.**
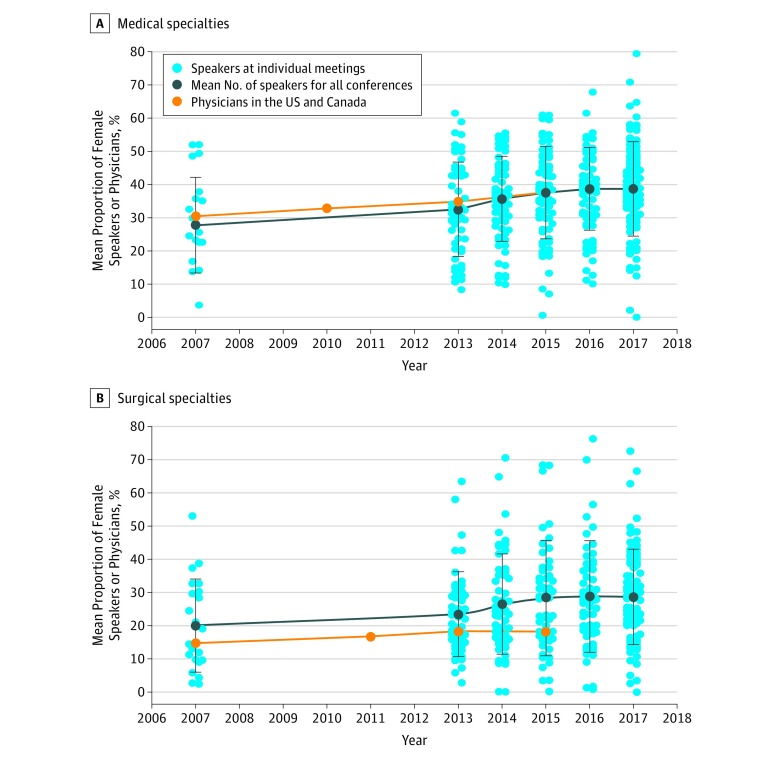
Mean Proportion of Female Speakers at Medical Specialty and at Surgical Specialty Conference Meetings per Year From 2007 to 2017 vs the Proportion of Female Physicians Practicing in Medical Specialties and Family Medicine or in Surgical Specialties, in the United States and Canada

Medical specialty conferences had a mean (SE) of 9.8% (1.9%) more female speakers than surgical specialty conferences for each year studied (*P* < .001). The mean (SD) proportion of female speakers at medical specialty conferences significantly increased from 29.9% (14.1%) in 2007 for 18 meetings to 38.8% (12.1%) for 100 meetings in 2017, with a range of 0% to 79.4% (*P* < .04) (Figure 3A). Between 2007 and 2015, the mean proportion of practicing physicians in medical specialties in the United States and in Canada who were female increased from 30.4% to 37.6%.^18,19,36,37,38^

The proportion of female speakers at surgical specialty conferences (including obstetrics and gynecology) also significantly increased (*P* < .02) between 2007 and 2017, from 20.1% (SD, 13.7%; 22 meetings) to 28.4% (SD 14.1%; 80 meetings) and ranged from 0.1% to 82.6% (Figure 3B). From 2007 to 2015, the mean proportion of female surgeons in the United States and in Canada increased from 14.8% to 18.3%.^18,19,36,37,38^ The mean proportions of conference speakers who were women were greater than the proportions of surgeons who were women for all years analyzed.

## Discussion

Low representation of female physicians in academia is an important aspect of gender inequity in medicine. Gender disparities in medical academia have been described for compensation, the granting process, authorship, and career advancement.^1,3,8,23,39^ Reports of gender disparities among conference speakers have been largely anecdotal.^28^ Previous work to characterize the gender gap in medical conferences has been limited by the size and scope of conferences included, nonsystematic inclusion of conferences, and lack of a validated strategy to assess the gender of speakers.^16,26^ Because conferences represent an important opportunity for role modeling, mentorship, and career advancement, understanding gender disparities in conference speakership is important for understanding gender inequity in medicine.^16^

We systematically identified medical conferences in Canada and in the United States and determined the proportion of female speakers at these conferences using a validated name-gender algorithm. In addition, we examined the trend during a 5-year period in the proportion of conference speakers who were women. Our results showed that women compose a minority of conference speakers, with a mean proportion of 34.1% for conferences held in 2017. Of the 701 meetings analyzed, only 82 meetings had more than 50% female speakers and 619 had less than 50% female speakers.

When framing these results in the context of existing gender demographics of physicians in Canada and the United States, we noted that the gap between the mean percentage of practicing physicians across all specialties who were female and conference speakers who were female decreased over time. In 2007, there was a 1.5% gap between female conference speakers and practicing female physicians in Canada and in the United States (26.1% compared with 24.6%). By 2015, the last year for which data on the gender of practicing physicians is available, there was a slightly higher proportion of conference speakers who were women than of practicing physicians who were women (32.7% compared with 32.4%).^18^

The increase in the proportion of female speakers in most specialties during the last decade has mirrored trends within the physician workforce.^18,19,36,37,38^ Although there is a lower proportion of female speakers at surgical specialty conferences than at medical specialty conferences, the mean proportion of conference speakers at surgical meetings who were female was greater than the mean proportion of practicing surgeons who were female. This finding suggests that the perceived gender gap in speakers at physician conferences likely represents the overall gender gap in academic medicine rather than a bias specific to conferences. This disparity is most obvious in surgical disciplines because only 25.9% of surgeons in the United States and in Canada were women in 2015.^18^ Low female visibility at these academic conferences may therefore be a product of low representation in surgical specialties.

Alternately, low representation of female physicians in prestigious academic positions, such as conference speakership, may contribute to low proportions of female physicians in select surgical and medical specialties.^18,19^ Although women have comprised more than 50% of medical school graduates since 1996 in Canada^40^ and have approached parity with male graduates in recent years in the United States,^41^ female physicians make up less than 50% of surgeons and academics.^3,18,19^ Low representation of female physicians in these opportunities signals to female medical graduates that these career tracks are not friendly to women and also contributes to lack of female mentorship.^6^

### Strengths and Limitations

The major strength of this study is that we used a validated tool, the GBAT, to assess the probability that a given first name is female rather than dichotomously assigning gender.^31,33^ Many first names have a degree of gender ambiguity; thus, previous studies that assigned gender dichotomously as male or female on the basis of the first name are subject to bias.^16,20,26^ Furthermore, our search strategy systematically identified a large number of academic conferences of different subspecialties; therefore, our results provided a more balanced representation of overall trends in academic medicine compared with previous publications that only assessed select meetings.^16,20,26^

Our study has some important limitations. The results did not account for conference size or importance because the proportions of female speakers at each conference were given equal weight when combining the results. We chose not to weigh conferences differentially because then smaller specialties could not be compared with larger specialties. Our result is therefore the mean proportion of speakers at each conference who were women and not the total proportion of all conference speakers who were women. In addition, the algorithm on which the GBAT is based has been shown to overestimate the proportion of women; however, this bias will affect all measured conferences equally and therefore should not influence our conclusions about the trend in female conference speakers over time.^33^ Owing to cultural, organizational, and structural factors that contribute to the gender gap and biases in medicine, our findings are not generalizable to conferences outside of North America or to nonmedical academic conferences.

The GBAT cannot assess representation of individuals belonging to different ethnic or racial backgrounds, does not capture how people self-identify their gender, and does not account for nonbinary genders.^31^ We acknowledge that women of additional visible minorities and nongender conforming physicians face more significant barriers and may be further underrepresented in medical conferences; this underrepresentation requires further assessment.

There was a wide range in the proportion of female speakers at medical conferences. On the basis of this result, we advocate for strategies to promote inclusivity in speaker invitation and selection for all conferences.^16,26,29^ We hypothesize that the low proportion of female speakers at medical conferences reflects broader gender inequity within the medical profession, particularly in subspecialties where the majority of physicians are men. It has been shown that the presence of female role models in male-dominated career streams can increase engagement of young women.^17^ Exposure to female speakers at medical conferences may be a means of encouraging female medical students and residents to choose specialties that have historically been male dominated. Strategies to promote inclusivity of female speakers at academic conferences may therefore represent an important opportunity to influence gender equity within medicine.

## Conclusions

To our knowledge, this is the first study to systematically identify medical conferences and to characterize the perceived gender inequities in conference speakership using a validated instrument. Our findings indicated that the proportions of female speakers at medical conferences increased during the last decade and that the gap between the proportion of female conference speakers and practicing physicians who were women decreased. Conference organizers should strive for diversity when inviting speakers.

## Notes

Supplement. **eAppendix.** Web of Science Search Terms **eTable 1**. Number and Mean Proportion of Female Speakers per Year of Conferences Divided by Specialty **eTable 2**. Specialties of Conferences Included in the Final Analysis
