# Supplementary material for: Trends in the Proportion of Female Speakers at Medical Conferences in the United States and in Canada, 2007 to 2017
Source: JAMA Netw Open. 2019 Apr 12;2(4):e192103. doi: 10.1001/jamanetworkopen.2019.2103 (PMC6481599; doi:10.1001/jamanetworkopen.2019.2103)
Supplement: Supplement. — eAppendix. Web of Science Search Terms eTable 1. Number and Mean Proportion of Female Speakers per Year of Conferences Divided by Specialty eTable 2. Specialties of Conferences Included in the Final Analysis [file jamanetwopen-2-e192103-s001.pdf]

Supplementary Online Content

Ruzycki SM, Fletcher S, Earp M, Bharwani A, Lithgow KC. Trends in the proportion of female speakers at medical conferences in the United States and in Canada, 2007 to 2017. *JAMA Netw Open*. 2019;2(4):e192103. doi:10.1001/jamanetworkopen.2019.2103

eAppendix. Web of Science Search Terms
eTable 1. Number and Mean Proportion of Female Speakers per Year of Conferences Divided by Specialty
eTable 2. Specialties of Conferences Included in the Final Analysis

This supplementary material has been provided by the authors to give readers additional information about their work.

© 2019 Ruzycki SM et al. *JAMA Network Open*.

eAppendix. Web of Science Search Terms

(cf=("Medicine" OR "Medic*" OR "Surg*" OR "Physician*" OR "Residen*" OR "Medical Student*" or "Patholog*" OR "Anesthesi*" OR "Cardiac Surg*" OR "Cardiolog*" OR "Immunolog*" OR "Allerg*" OR "Colorectal" OR "Critical Care" OR "Intensive Care" OR "Intensiv*" OR "Dermatolog*" OR "Diagnostic Radiolog*" OR "Radiolog*" OR "Emergen*" OR "Endocrinolog*" OR "Gastroenterolog*" OR "Gynecolog*" OR "General Surg*" OR "Geriat*" OR "Hematolog*" OR "Infectio*" OR "Internal*" OR "Geneti*" OR "Medical Microbiolog*" OR Neonatal*" OR "Nephrolog*" OR "Neurolog*" OR "Neurosurg*" OR "Nuclear Med*" OR "Obstetric*" OR "Opthalmolog*" OR "Orthoped*" OR "Otolaryngolog*" OR "Head and Neck" OR "Pediatr*" OR "Physiat*" OR "Physical Medic*" OR "Plastic Surg*" OR "Psychiatr*" OR Radiaton*" OR "Respirolog*" or "Pulmon*" OR "Rheumatolog*" OR "Thoracic" OR "Urolog*" OR "Vascular") NOT cf=("Europ*" NOT "Asia*" NOT "Australia*" NOT "China" NOT "Chinese" NOT "India*" NOT "British" NOT "German" NOT "Veterinary" NOT "Swiss" NOT "Japan" NOT "Britain" NOT "Engineering" NOT "Nurse*" NOT "International" NOT "Mexic*")) *AND* **LANGUAGE:** (English)

© 2019 Ruzycki SM et al. *JAMA Network Open*.

eTable 1. Number and Mean Proportion of Female Speakers per Year of Conferences Divided by Specialty

Specialty	**2007**	**2013**	**2014**	**2015**	**2016**	**2017**	**Change**
Anesthesia (n=12)	19.61	24.94	25.19	26.01	27.01	29.88	1.52
Dermatology (n=4)	51.93	41.66	38.06	44.20	31.55	39.88	0.77
Emergency medicine (n=3)	16.83	17.76	26.57	32.26	29.57	34.10	2.02
Family medicine (n=3)	51.97	58.88	54.62	47.39	55.86	46.66	0.90
Medical genetics (n=1)	n/a	n/a	n/a	48.23	47.43	45.52	
Internal Medicine (n=46)	18.22	33.78	32.08	33.85	36.22	34.57	1.90
Allergy (n=2)	14.14	23.69	44.14	0.56	39.50	38.46	
Cardiology (n=10)	n/a	25.18	19.46	19.78	17.75	18.48	
Endocrinology (n=1)	n/a	n/a	n/a	43.44	39.79	57.57	
Geriatrics (n=2)	n/a	51.34	53.69	55.42	50.28	51.18	
Gastroenterology (n=2)	n/a	22.09	19.35	n/a	27.55	33.38	
Hematology (n=3)	n/a	n/a	n/a	n/a	n/a	29.20	
ICU (n=2)	24.51	27.93	31.54	31.40	33.30	29.80	

© 2019 Ruzycki SM et al. *JAMA Network Open*.

ID (n=9)	3.63	33.81	36.11	37.00	39.02	40.63	
Nephrology (n=1)	n/a	n/a	28.95	28.16	27.90	34.84	
Oncology (n=4)	25.55	33.05	22.58	47.14	42.39	34.27	
Respirology (n=1)	n/a	n/a	n/a	n/a	13.77	20.08	
Rheumatology (n=3)	n/a	50.06	40.68	44.46	48.86	38.52	
Other (n=5)	23.27	55.00	33.44	21.66	43.60	42.04	
Neurology (n=4)	n/a	28.48	33.44	32.90	32.70	37.57	1.32
Neurosurgery (n=2)	n/a	11.61	26.09	48.08	25.16	18.15	1.56
Obstetrics and gynecology (n=13)	36.58	38.49	46.19	51.73	54.71	48.78	1.33
Ophthalmology (n=7)	n/a	23.78	29.60	35.10	25.03	34.57	1.45
Orthopedic surgery (n=4)	n/a	5.84	12.22	10.70	10.39	10.67	1.83
Pathology (n=5)	49.32	28.38	34.59	50.50	35.89	45.28	0.92
Pediatrics (n=4)	n/a	23.92	38.50	31.91	35.89	37.96	1.59
Plastic Surgery (n=9)	21.65	23.77	25.70	23.99	29.55	28.46	1.31
Psychiatry (n=6)	n/a	49.84	50.01	48.58	49.36	51.80	1.04

© 2019 Ruzycki SM et al. *JAMA Network Open*.

Physiatry (n=1)	n/a	n/a	n/a	n/a	n/a	40.97	
Radiation Oncology (n=1)	n/a	31.32	36.82	35.22	40.46	34.53	1.10
Radiology (n=9)	36.18	39.63	37.21	38.17	34.40	37.37	1.03
Surgery (n=24)	19.46	24.18	26.08	27.24	27.18	28.90	1.49
Urology (n=3)	9.47	19.57	25.84	20.73	25.78	21.81	2.30
Vascular Surgery (n=9)	14.76	15.88	23.16	21.97	22.63	19.01	1.29
Other (n=8)	n/a	33.05	33.93	35.31	44.17	44.08	1.33

© 2019 Ruzycki SM et al. *JAMA Network Open*.

eTable 2. Specialties of Conferences Included in the Final Analysis

Specialty	No. of Meetings
Surgical	81
General surgery	24
Neurosurgery	2
Obstetrics and gynecology	13
Ophthalmology	7
Orthopedic surgery	4
Plastic surgery	9
Urology	3
Other or combined surgical disciplines	19
Medical	100
Anesthesia	12
Dermatology	4
Emergency medicine	3
Family medicine	3
Medical genetics	1
Internal medicine and subspecialties	46
Neurology	1
Pathology	5
Pediatrics	4
Psychiatry	6
Physiatry	1
Radiation oncology	1
Radiology	9
Other medical or combined disciplines[a]	4

[a]Includes medical education, wilderness medicine, aerospace medicine, and combined disciplines (such as cardiology-cardiac surgery and transplant medicine).

© 2019 Ruzycki SM et al. *JAMA Network Open*.
